# Supplementary material for: Genetic and epigenetic variation in the lineage specification of regulatory T cells
Source: eLife. 2015 Oct 28;4:e07571. doi: 10.7554/eLife.07571 (PMC4623597; doi:10.7554/eLife.07571)
Supplement: Supplementary file 14. — Autoimmune and metabolic phenotypes genome-wide association (GWA) studies analyzed. DOI: http://dx.doi.org/10.7554/eLife.07571.027 [file elife07571s014.docx]

We analyzed diseases phenotype GWA studies for aggregated autoimmune and metabolic disease sets. Names are identical to study description in NHGRI GWAS catalog.

We analyzed GWA studies for the following autoimmune disease phenotypes:
Multiple sclerosis

Crohn's disease

Ulcerative colitis

Type 1 diabetes

Rheumatoid arthritis

Systemic lupus erythematosus

Asthma

C-reactive protein

Celiac disease

Age-related macular degeneration

Immune response to smallpox vaccine (IL-6)

Psoriasis

Immune reponse to smallpox (secreted IFN-alpha)

Response to tocilizumab in rheumatoid arthritis

Systemic sclerosis

Ankylosing spondylitis

Inflammatory biomarkers

Atopic dermatitis

Type 1 diabetes autoantibodies

Neutrophil count

White blood cell types

Tuberculosis

Immune reponse to smallpox (secreted IL-2)

IgE levels

Celiac disease and Rheumatoid arthritis

Inflammatory bowel disease

Immune reponse to smallpox (secreted IL-1beta)

Immunoglobulin A

Behcet's disease

Type 1 diabetes nephropathy

Multiple sclerosis (severity)

Immune reponse to smallpox (secreted IL-12p40)

Complement C3 and C4 levels

Response to TNF antagonist treatment

IgE grass sensitization

Cytomegalovirus antibody response

IgA nephropathy

C-reactive protein and white blood cell count

Alopecia areata

Allergic rhinitis

Age-related macular degeneration (GA)

Response to TNF-alpha inhibitors in rheumatoid arthritis

Multiple sclerosis (age of onset)

Immune response to anthrax vaccine

Asthma (childhood onset)

Immune reponse to smallpox (secreted IL-10)

Immune reponse to smallpox (secreted TNF-alpha)

Arthritis (juvenile idiopathic)

Age-related macular degeneration (CNV)

We analyzed GWA studies for the following metabolic disease phenotypes:
Type 2 diabetes

Coronary heart disease

HDL cholesterol

Body mass index

LDL cholesterol

Triglycerides

Metabolite levels

Phospholipid levels (plasma)

Blood pressure

QT interval

Cholesterol, total
